# Prognostic Value of Perineural Invasion in Gastric Cancer: A Systematic Review and Meta-Analysis

**DOI:** 10.1371/journal.pone.0088907

**Published:** 2014-02-21

**Authors:** Jing Deng, Qihan You, Yang Gao, Qing Yu, Peng Zhao, Yulong Zheng, Weijia Fang, Nong Xu, Lisong Teng

**Affiliations:** 1 Department of Medical Oncology, The First Hospital Affiliated to Medical School of Zhejiang University, Hangzhou, China; 2 Department of Pathology, The First Hospital Affiliated to Medical School of Zhejiang University, Hangzhou, China; 3 Medical School of Zhejiang University, Hangzhou, China; 4 Department of Surgical Oncology, The First Hospital Affiliated to Medical School of Zhejiang University, Hangzhou, China; University of Nebraska Medical Center, United States of America

## Abstract

**Background:**

The prognostic role of perineural invasion in gastric cancer is controversial. Here, we present a systemic review and meta-analysis of the association between perineural invasion and survival in resectable gastric cancer patients.

**Methods:**

A comprehensive literature search for relevant reports published up to April 2013 was performed using PubMed, Embase, Web of Science and Wanfang Data. Studies that investigated the role of perineural invasion with a sample size greater than 100 were included and analyzed.

**Results:**

A total of 30,590 gastric cancer patients who had undergone curative gastrectomy from twenty-four studies were included. The median rate of perineural invasion positive was 40.9% (6.8%–75.6%). Fourteen studies investigated overall survival unadjusted for other variables in 23,233 gastric cancer patients. The relative hazard estimates ranged from 0.568–7.901 with a combined random effects estimate of 2.261 (95% CI = 1.841–2.777, P = 0.000). The effect of perineural invasion on overall survival adjusted for other prognostic factors was reported in 17 studies incorporating 8,551 cases. The hazard estimates ranged from 0.420–8.110 with a pooled random effects estimates of 1.484 (95% CI = 1.237–1.781, P = 0.000). There was heterogeneity between the studies (Q = 49.22, I-squared = 67.5%, P = 0.000). Disease-free survival was investigated adjusted in four studies incorporating 9,083 cases and the pooled fixed hazard ratio estimate was 1.371(95% CI = 1.230–1.527, P = 0.000).

**Conclusion:**

Perineural invasion is an independent prognostic factor affecting overall survival and disease-free survival of gastric cancer patients who had undergone the curative resection. This effect is independent of lymph node status, tumor size and the depth of invasion as well as a range of other biological variables on multivariate analysis. Large prospective studies are now needed to establish perineural invasion as an independent prognostic marker for gastric cancer.

## Introduction

Gastric cancer is the fourth most common cancer worldwide and also the second leading cause of cancer-related death in Asia. Although underwent radical resection, most of gastric cancer patients will die of recurrence and metastasis, with 5-year overall survival no more than 50% for resectable patients in China [1].

Perineural invasion (PNI) is the process of neoplastic invasion of nerves and is an under-recognized route of metastatic spread [2]. Up to now, the research of PNI pathogenesis is still in its infancy. However, PNI is found to be related to a more aggressive tumor phenotype and poor prognosis in several malignancies, most notably head and neck and prostate cancers.

In gastric cancer, the prognostic significance of PNI had been investigated in a few studies, but they had not reached consensus. Ahmet Bilici found that the median survival of PNI-positive patients is much shorter than that of PNI- negative ones and demonstrated that PNI is a useful prognostic factor for curative gastric cancer [3]. However, in the study of Duraker, although the positivity of PNI is 59.6% and with the progression of gastric carcinoma, the incidence of PNI increased, PNI did not provide any additional prognostic information to the classical parameters [4].

The aim of our study was to evaluate the prognostic role of PNI in gastric cancer by systematically reviewing the available evidence. We identified all published reports that assessed the relationship between PNI and outcome in gastric cancer and performed a meta-analysis using standard statistical techniques. A protocol was developed a priori from the research question – ‘Is PNI an independent prognostic factor affecting survival of gastric cancer patients who had undergone the curative gastric resection?’.

## Methods

We have adhered to the recommendations of the Preferred Reporting Items for Systematic Reviews and Meta-Analyses (PRISMA) statement [5]. The checklist of items included in meta-analysis was available in checklist S1. Inclusion criteria and methods of the analyses were specified in advance.

### Literature Search Strategy

We searched electronic databases PubMed, Embase, Web of Science and Wanfang Data for studies to include in this system review and meta-analysis. The upper date limit of April 30, 2013 was applied, with no lower date limit. Articles published in English and Chinese were searched. A review of reference lists was also performed. Searches include the terms “gastric cancer perineural invasion” and search details are (“stomach neoplasms”[MeSH Terms] OR (“stomach”[All Fields] AND “neoplasms”[All Fields]) OR “stomach neoplasms”[All Fields] OR (“gastric”[All Fields] AND “cancer”[All Fields]) OR “gastric cancer”[All Fields]) AND perineural [All Fields] AND invasion [All Fields].

### Eligibility Criteria

To be eligible for inclusion in this system review and meta-analysis, a study must: (1) investigate the association of PNI with overall survival (OS) or disease-free survival (DFS) of gastric cancer patients who had undergone curative gastrectomy. (2) assess the PNI in gastric cancer tissue obtained by surgical resection. (3) report HR or with sufficient data to estimate the HR. (4) include more than 100 patients. Reviews, poor quality articles or articles that have repeated data from the same population were excluded,. Abstracts of all candidate articles were read by two independent readers (JD and QHY). Articles that could not be categorized based on title and abstract alone were retrieved for full-text review. Disagreements were resolved by consensus between the two readers.

### Data Extraction and Quality Assessment

Two reviewers (JD and QHY) independently extracted the data of the included studies. The following information was extracted from each included study: study characteristics (first author, year of publication, study design, inclusion criteria, number of patients, follow-up time), patient characteristics (age, gender, rate of lymph node metastasis, PNI positive rate etc.), treatment characteristics (surgery, chemotherapy and radiotherapy), study design (PNI detection method), statistical analysis (Univariate and/or Multivariate analysis statistic data). Study quality was assessed independently by two researches (JD and QHY) according to the Newcastle-Ottawa quality assessment scale [6].

### Statistical Analyses

Meta-analysis was performed by using the DerSimonian – Laird random effects model [7].Statistical analyses were carried out using Stata version 11.0 (Stata corp LP. Texas. USA.). Pooled estimates of the HRs were obtained by fixed-effect or random-effects meta-analysis according to heterogeneity using the inverse-variance weighting method based on published confidence intervals for the HRs. For those studies that did not report the HR but did provide sufficient information on survival by PNI status, we estimated the HR and confidence intervals according to the method of Parmer et al [8].

Q statistic test and I-squared statistic were used to estimate the heterogeneity of these studies, if P>0.05, it means studies had little heterogeneity and fixed-effect model (inverse variance) could be used in statistic analysis; if these studies had great heterogeneity, the cause of heterogeneity was analyzed [9], [10]. An I-squared value>50% was considered to represent substantial heterogeneity between studies, I-squared value <70% was considered that heterogeneity between studies could be accepted [11]. Publication and selection bias were investigated through a funnel plot by Egger’s and Begg’s test [12], [13].

## Results

### Literatures Information

**Figure 1:**
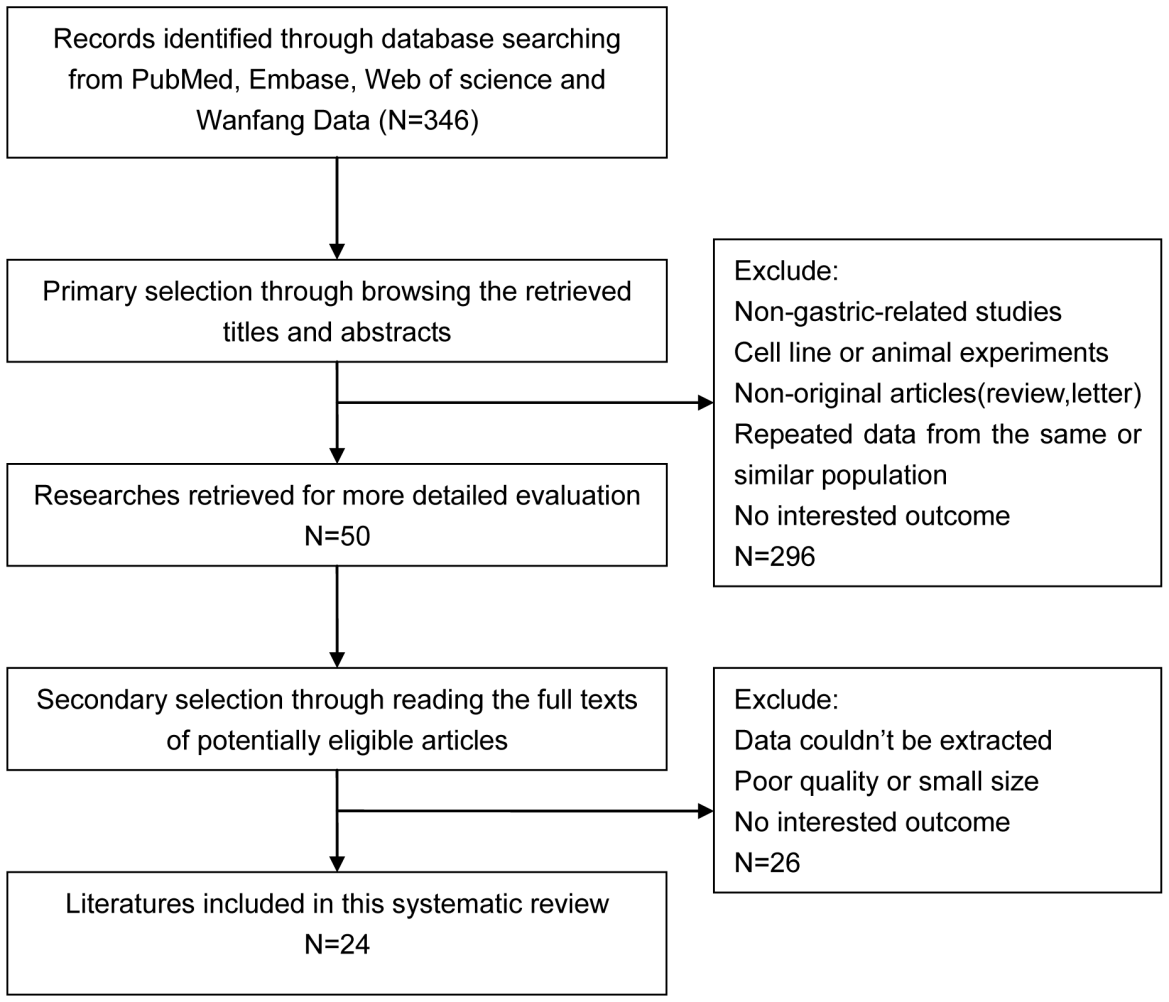
Flow diagram of study selection procedure.

Three hundred and forty-six articles were identified initially using the search strategy above. Two hundred and ninety-six of those were excluded due to non-gastric-related studies, non-original articles (review, letter), or having repeated data from the same population through reading titles and abstracts. After reading full texts of potentially eligible articles, those which are small sized or poor quality or have no related outcome or data couldn’t be extracted were excluded and finally 24 studies were included in this systemic review and meta-analysis [3], [4], [14]–[35](figure 1).

### Study Characteristics

In the 24 included studies, Seventeen were based on Asian population [3], [4], [14], [17], [21]–[29], [31]–[33], [35], five were from Europe [15], [16], [18]–[20] and the other two were from America [30], [34]. A total of 30,509 patients with a median of 319.5 (ranged from 142 to 10728) were included, about two thirds were male patients. The rate of lymph node metastasis was 62.1% (0–83.04%). A median of 40.9% (6.8%–75.6%) patients were PNI positive. Twenty-two studies used light microscopy hematoxylin and eosin (HE) staining to judge PNI, two used light microscopy laminin staining. PNI was assessed as positive when cancer cells were seen in the perineurium or neural fascicles intramurally. With regards to treatments, eleven of studies used surgery only, nine studies used surgery with or without adjuvant chemotherapy, while the other four studies used surgery with or without adjuvant chemotherapy and radiotherapy. The Characteristics of the included studies were summarized in table S1 and the statistical analysis data in table S2.

### Systemic Review and Meta-analysis

Fourteen studies [14], [15], [17], [19], [22]–[25], [28]–[31], [32], [35] including 23,233 patients reported the effect of PNI on OS in univariate analyses ranging from 0.568–7.901. Thirteen articles supported PNI was a prognostic factor for OS in gastric cancer patients who had undergone curative resection. One article from Spain did not draw this conclusion. The pooled random effects estimate was 2.261 (95% CI = 1.841–2.777, P = 0.000) which demonstrated PNI was an independent factor influencing OS of gastric cancer. However, there was evidence for significant heterogeneity between the studies (P = 0.000). There was no evidence for publication bias (P = 0.913 in Begg’s Test, P = 0.469 in Egger’s test).

**Figure 2:**
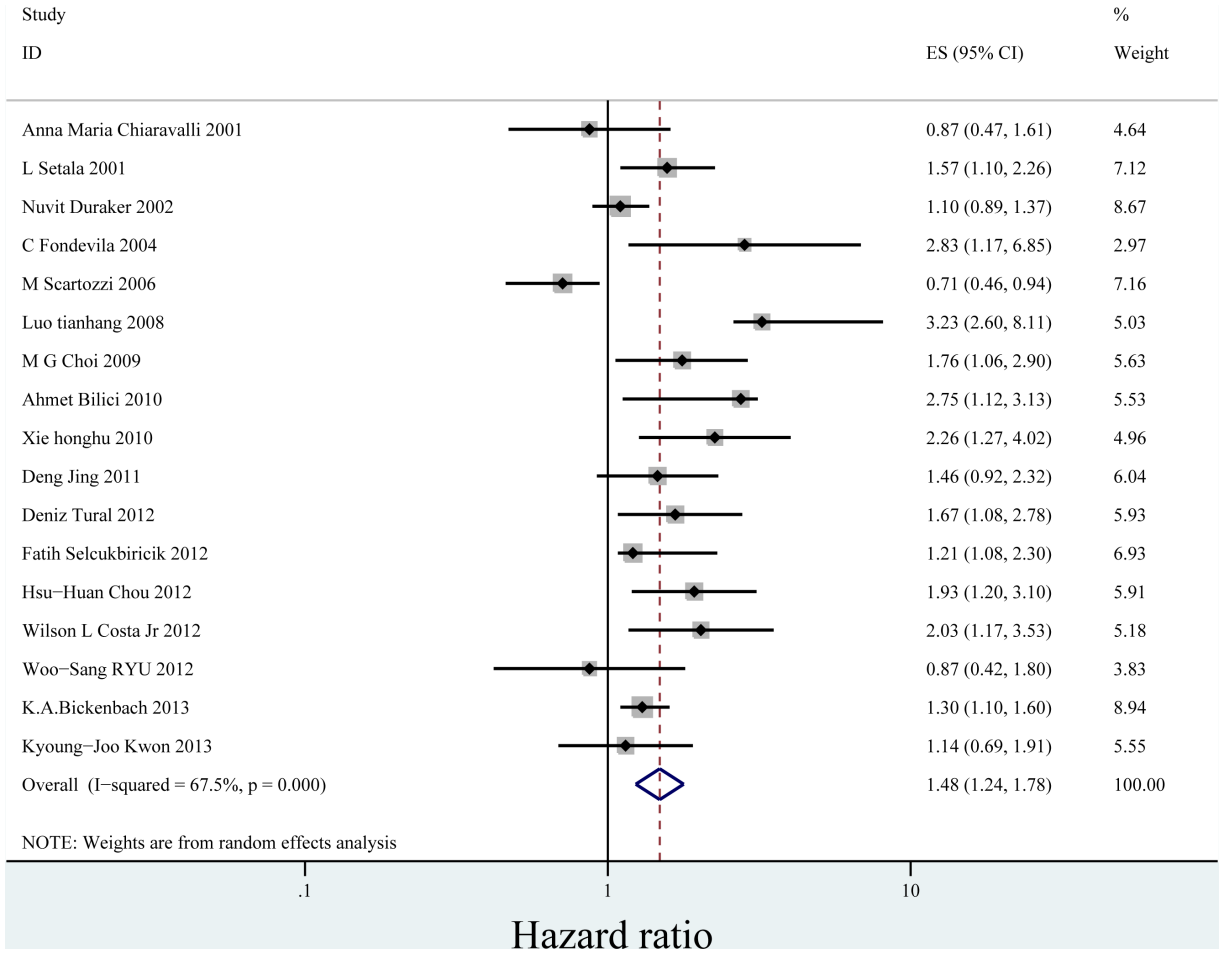
Forrest plot of combined hazard ratio for the association of PNI and OS in multivariate analysis.

**Figure 3:**
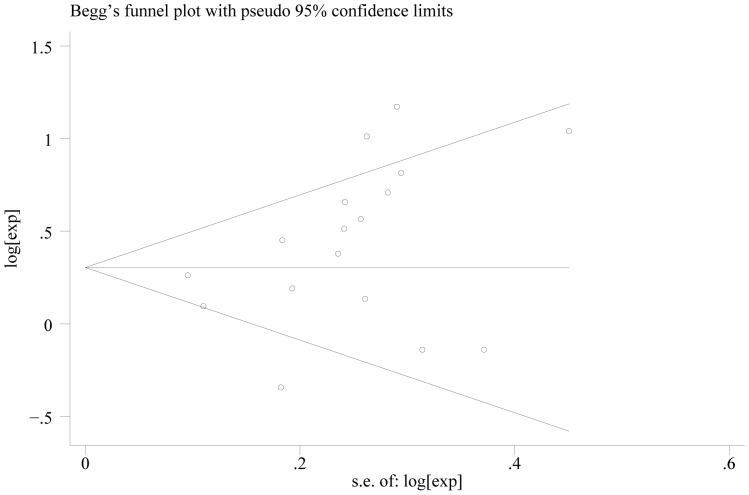
Begg’s test result of OS.

In the seventeen studies [3], [4], [16], [18]–[23], [25]–[28], [30], [31], [34], [35] reported the effect of PNI on OS in multivariate analysis for other risk factors, eleven supported PNI was an independent factor while the other six did not. Seventeen studies including 8,551 patients reported the effect of PNI on OS in analyses adjusted for other risk factors. The adjusted HR estimates for OS from these studies ranged from 0.460–8.110. The pooled random effects estimate was 1.484 (95% CI = 1.237–1.781, P = 0.000), which demonstrated PNI was an independent factor influencing OS of gastric cancer who had undergone curative surgery (Figure 2). There was heterogeneity between the studies (Q = 49.22, I-squared = 67.5%, P = 0.000). No evidence for publication bias (P = 0.064 in Begg’s Test, P = 0.078 in Egger’s test) was detected (Figure 3).

**Figure 4:**
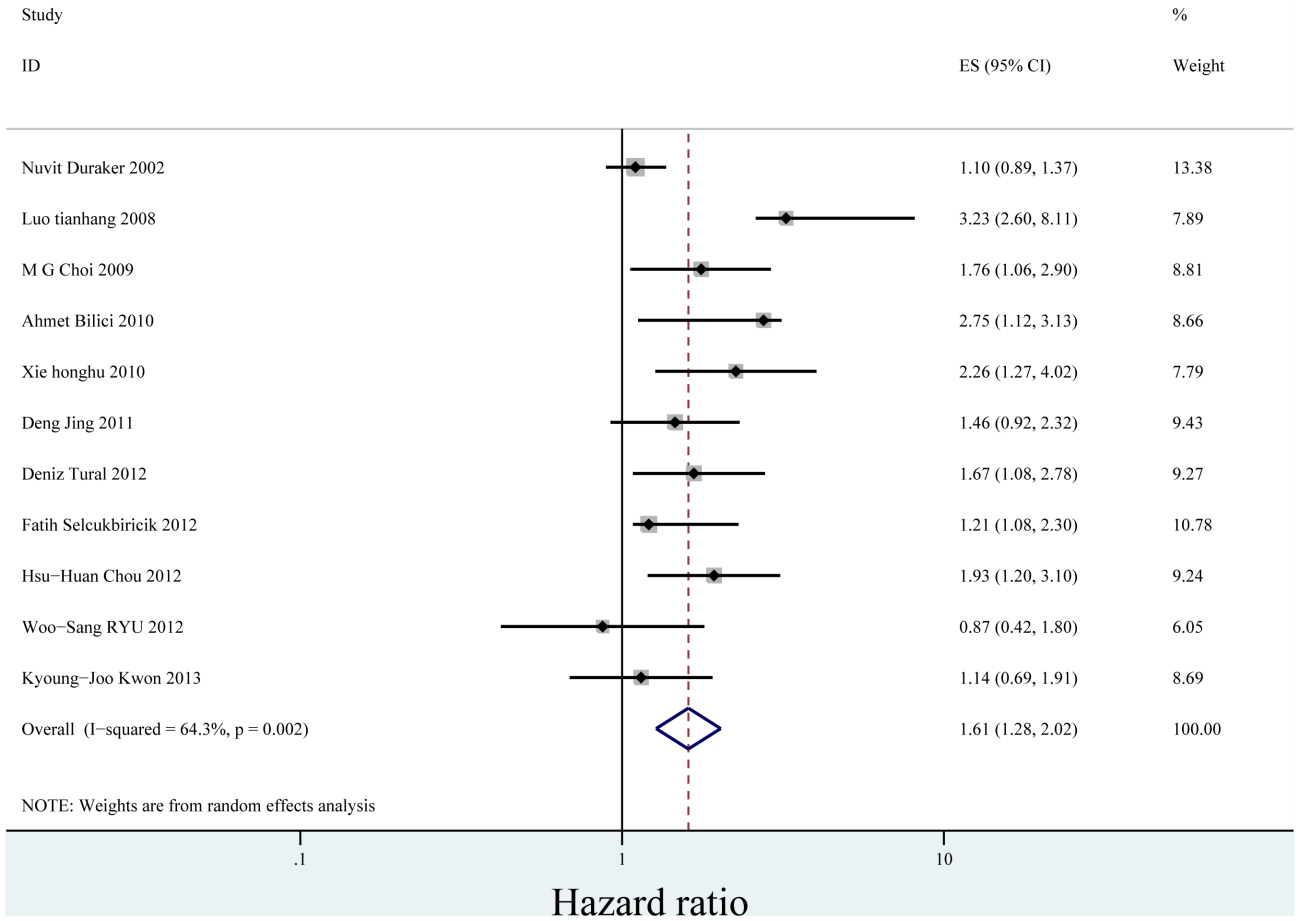
Forrest plot of combined hazard ratio for the association of PNI and OS of Asian in multivariate analysis.

**Figure 5:**
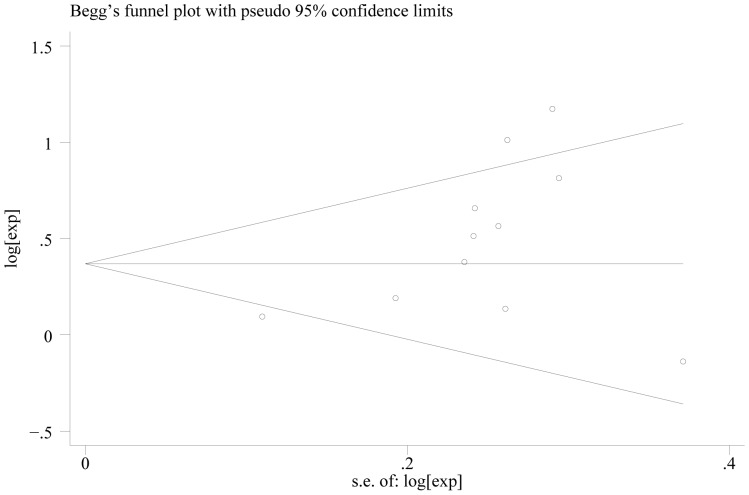
Begg’s test result of OS in Asian.

**Figure 6:**
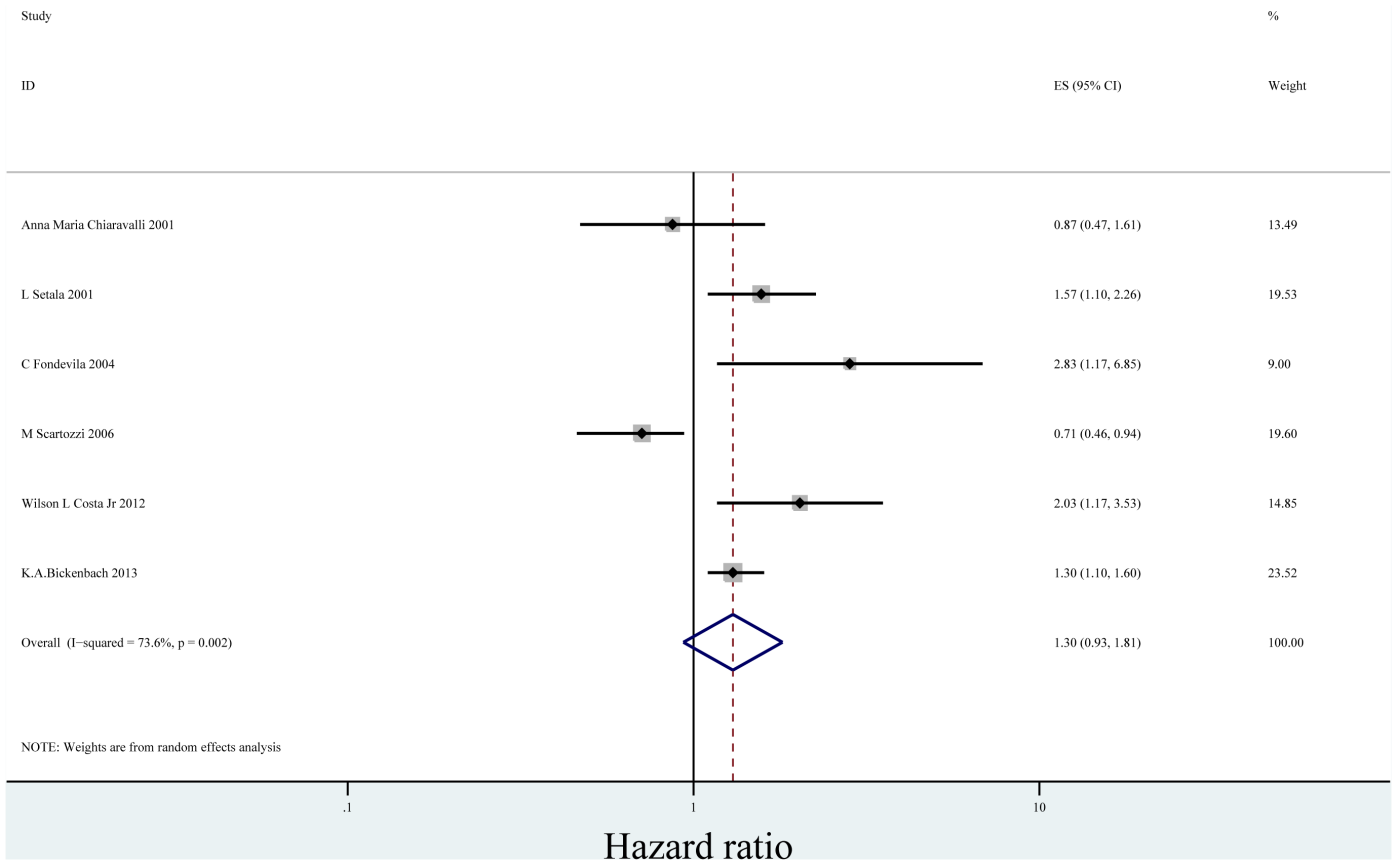
Forrest plot of combined hazard ratio for the association of PNI and OS of non-Asian in multivariate analysis.

**Figure 7:**
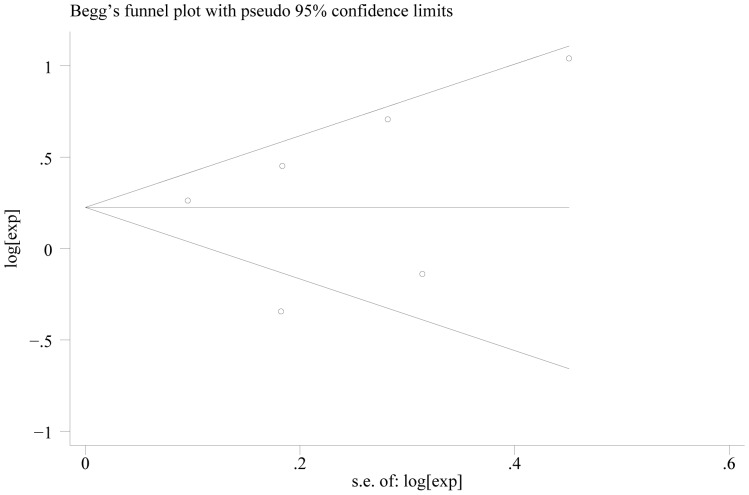
Begg’s test result of OS in non-Asian.

When we stratified the studies by ethnicity, the pooled HR for Asian (eleven studies) [3], [4], [21]–[23], [25]–[28], [31], [35] was1.606 (95% CI = 1.275–2.024)(P = 0.000) (Figure 4). There was heterogeneity between the studies (Q = 28.04, I-squared = 64.3%, P = 0.002). No evidence for publication bias (P = 0.087 in Begg’s Test, P = 0.052 in Egger’s test) was detected (Figure 5). The pooled HR for patients in non-Asian (six studies from Europe and America) [16], [18]–[20], [30], [34] was 1.300 (95% CI = 0.935–1.808)(P = 0.119) (Figure 6). Heterogeneity was found in this group (Q = 18.96, I-squared = 73.6%, P = 0.002). There was no evidence for publication bias (P = 0.26 in Begg’s Test, P = 0.747 in Egger’s test) (Figure 7). There exists significant difference between Asian and non-Asian. It means PNI independently affected OS of Asian gastric cancer patients while this effect has no statistically significance in non-Asian patients.

**Figure 8:**
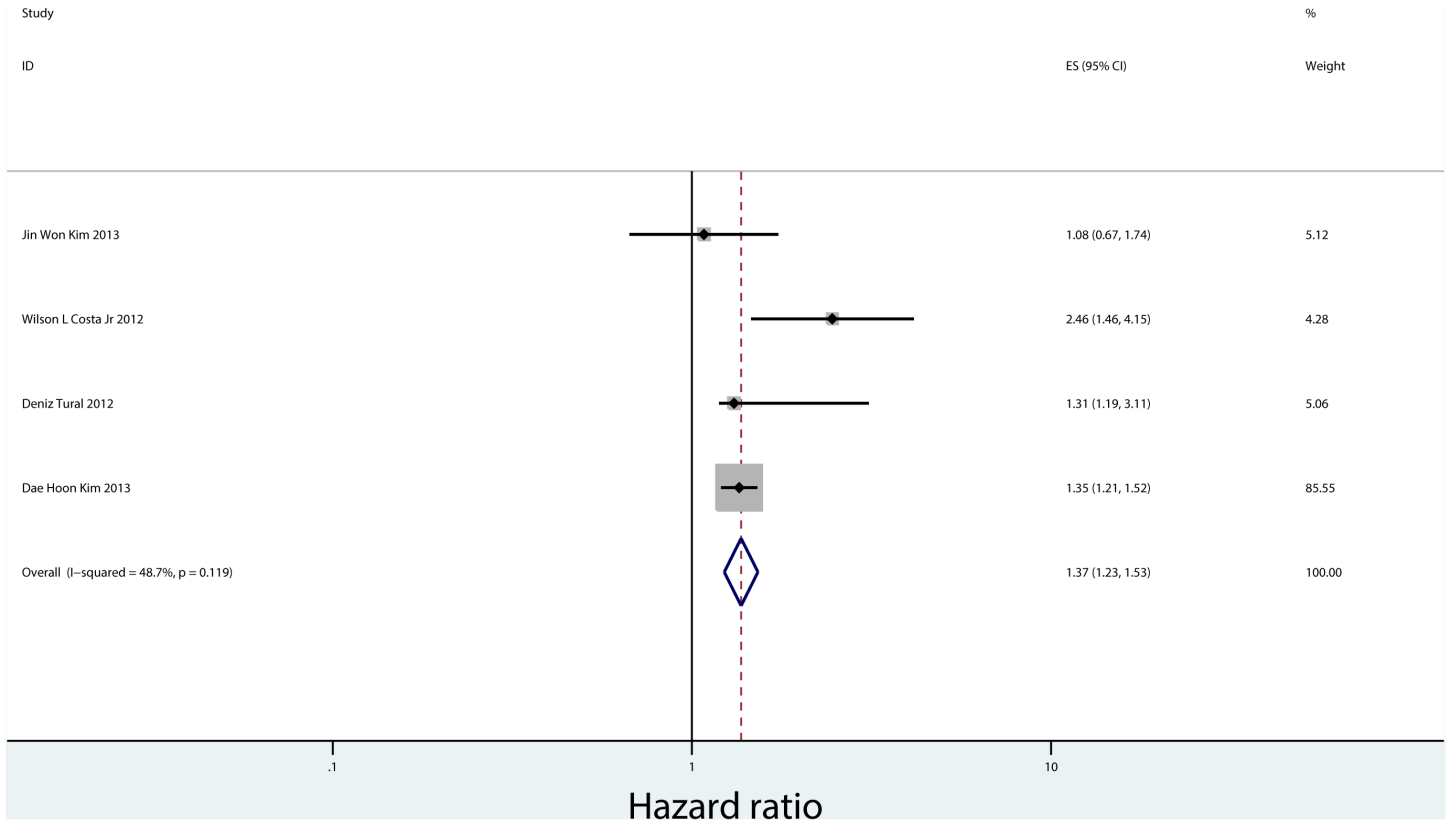
Forrest plot of combined hazard ratio for the association of PNI and DFS in multivariate analysis.

**Figure 9:**
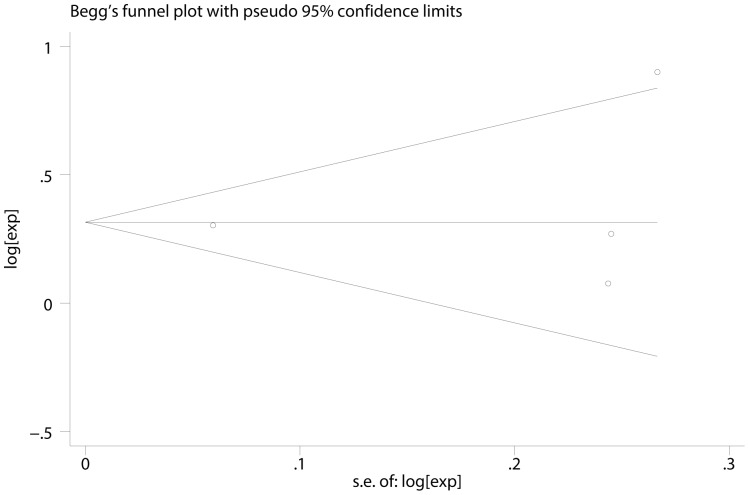
Begg’s test result of DFS.

There were only four studies that reported the effect of PNI on DFS, which include 9,083 patients from Korea, Brazil and Turkey [26], [30], [32], [33]. Two of these studies reported the relationship between PNI and DFS in both univariate analysis and multivariate analysis, and the other two reported that only in multivariate analysis. The pooled fixed HR in multivariate analysis was 1.371(95% CI = 1.230–1.527, P = 0.000), indicating PNI is an independent prognostic factor for gastric cancer recurrence(Figure 8). There was no substantial heterogeneity between the studies (Q = 5.85, I-squared = 48.7%, P = 0.119). There was no evidence for publication bias (P = 0.308 in Begg’s Test, P = 0.721 in Egger’s test) (Figure 9).

Seven cohorts also reported the data of lymph node metastasis, depth of tumor invasion and PNI in gastric cancer. The combined HRs showed that PNI was significantly associated with lymph-node metastasis (HR: 1.322, 95% CI: 1.249–1.400, P = 0.000) and depth of tumor invasion (HR: 1.652, 95% CI: 1.561–1.748, P = 0.000). There were only three studies presented the data on PNI and vascular invasion. Further, combined HR of 2.482 (95% CI: 2.056–2.995, P = 0.000) indicated that PNI was associated with tumor vascular invasion in gastric cancer. However, PNI seems not affected by age, sex and tumor location.

## Discussion

PNI is a marker of poor outcome and a harbinger of decreased survival in many malignancies, such as pancreatic cancer, prostate cancer and head and neck cancer [2]. In gastric cancer, studies are conflicting regarding prognostic significance of PNI. The presence of both significant and non-significant studies addressing the importance of PNI in gastric cancer made it necessary to perform a quantitative aggregation of the survival results. This systematic review and meta-analysis incorporated 30,590 cases significantly demonstrate the independent prognostic role of PNI (adjusted pooled HR estimates of 1.484) in gastric cancer showing that this effect is independent of lymph node status, tumor size and tumor grade as well as a range of other biological variables on multivariate analysis. PNI is as well a predictor for recurrence of gastric cancer patients who had undergone curative resection. Therefore, we advocate PNI status should be considered for gastric cancer post-surgery therapy stratification and weighed together with other known adverse tumor features. Compared to PNI negative ones, we recommend more active therapy be given to PNI positive patients.

Most of cohorts in this review are from Asia. As we know, the incidence of gastric cancer in Asian countries is much higher than that in western countries because of difference of genetic inheritance and dietary habit. Our study found that PNI significantly affected the survival of Asian gastric patients. However, in non-Asian patients, although there is a trend that presence of PNI predicts shortened OS, this effect is not statistically significant. Since only six cohorts are from Europe and America, more data should be collected to elucidate the role of PNI in prognosis.

We also conducted a pooled analysis on the relationship of PNI and other tumor characteristics, demonstrating PNI was significantly related to lymph node metastasis, depth of tumor invasion and vascular invasion. Just as T stage and N stage, PNI is an important marker of cancer invasiveness.

This meta-analysis is, to our knowledge, the first study which systematically estimates the association between PNI and the prognosis of gastric cancer patients who had undergone radical surgery. The finding of study heterogeneity in HR estimates is unsurprising. And the heterogeneities in this meta-analysis are within the accepted limits. Different cohorts and ethnicity of patients were used. Patients were in different period and received different types of surgery. And furthermore, the positivity of PNI might be affected by amount of tissue obtained, biopsy technique, histological section, number of times tissue section taken for examination and inter-observer variations. In order to acquire consistence and reproducibility among the inter-observer studies and minimize subjectivity, accurate identifying PNI is very important. In this systematic review, most studies used light microscopy, HE staining to observe perineural invasion. PNI was assessed as positive when cancer cells were seen in the perineurium or neural fascicles intramurally by pathologists. However, we found Leibig’s definition on PNI is more precise and operable. She defined PNI as the presence of cancer cells along nerves and/or within the epineurial, perineurial and endoneurial spaces of the neuronal sheath, including cases in which the cells circumscribed at least 33% of the nerve [2]. This definition, we thought, could be a uniform criterion in judging PNI in the light microscope with HE staining in gastric cancer in the future. In addition, the minimum amount of tissue obtained and number of tissue section observed should be set to guarantee the accuracy of PNI judgment.

In spite of this, in some case it is difficult to identify the perineurium accurately on a HE-stained glass slide, using S100 immunohistochemical staining is by far the most classic method for detecting PNI. And a recent immunostaining technique for identifying perineurium using antibodies such as glucose transporter protein 1 (Glut1) has revealed that tumor nests are often located outside the perineurium in a typical PNI focus [36]. These immunostaining techniques should be used to identify PNI status in the future studies which focus on the relationship of PNI and prognosis.

The effect of bias on the meta-analysis should also be considered. In our study, we excluded studies using small series (less than 100 patients) and those that did not provide minimum data for the pooled analysis. The exclusion of small studies may have minimized the effect of publication bias – the non-publication of studies with null results – by not including reports of small series that are more likely to be published if they show a positive result. As we know, HRs reported by smaller studies were systematically larger than those reported by the larger studies. The inclusion of only large studies and those that meet minimum quality criteria in the meta-analysis maximised the chance of the pooled estimate of HR representing the true HR [37]. In our study, we did not detect a substantial degree of publication bias, indicating no large negative studies have been missed in our literature search. We used two most-used languages–English and Chinese to minimize language bias. What’s more, a selection process with rigid inclusion criteria was adopted in ascertaining studies, thereby reducing selection bias.

In conclusion, PNI is an underreported phenomenon in gastric cancer. This study strongly suggest that PNI could function as an independent prognostic factor affecting OS and DFS in curative gastric cancer patients and support consideration of PNI status for gastric cancer therapy stratification. Large-scale and well-designed prospective cohort studies are necessary to validate our findings in the future.

## Supporting Information

Table S1 Characteristics of the studies.
(DOCX)

Table S2 Statistical analysis data for OS of the studies.
(DOCX)

Checklist S1 Checklist of items included in meta-analysis.
(DOCX)
