# Supplementary material for: Prognostic Value of Perineural Invasion in Gastric Cancer: A Systematic Review and Meta-Analysis
Source: PLoS One. 2014 Feb 21;9(2):e88907. doi: 10.1371/journal.pone.0088907 (PMC3931634; doi:10.1371/journal.pone.0088907)
Supplement: Table S1 — Characteristics of the studies. (DOCX) [file pone.0088907.s001.docx]

**Table S1.** Characteristics of the included studies.

| Author | Year | Country | Male (%) | Age | N | Follow-up time(months) | LN(+) rate(%) | PNI detectionMethod | PNI positive rate(%) | Treatment |
| --- | --- | --- | --- | --- | --- | --- | --- | --- | --- | --- |
| Akira Tanaka[14] | 1993 | Japan | 58.3 | 59(23-89) | 283 | 59(23-89) | 83.04 | Laminin | 49.1 | S |
| L Setala[15] | 1996 | Finland | 55.8 | 67.6(22.9-93) | 321 | 10.2(4.2-17.9) | 46.09 | HE | 46.4 | S |
| Anna M Chiaravalli[16] | 2001 | Italy | NA | NA | 185 | >60 | 72.43 | HE | NA | S |
| Chia-Siu Wang[17] | 2001 | Taibei | 55.8 | 63.4(14-92) | 1322 | 42.8 | 62.1 | HE | 38.58 | S±C |
| L Setala[18] | 2001 | Finland | 58 | 65.9±12.2* | 198 | 120±45.6* | 53.03 | HE | 48 | S |
| Nuvit Duraker[4] | 2002 | Turkey | 70.3 | 56.7(23-79) | 354 | NA | 72.6 | HE | 59.6 | S |
| C Fondevila[19] | 2004 | Spain | 63.0 | 67±12 | 156 | 43(37-49) | 57.59 | HE | 12.8 | S±C |
| M Scartozzi[20] | 2006 | Italy | 60.1 | 68(30-94) | 734 | >60 | 58.99 | HE | 6.8 | S |
| Luo tianhang[21] | 2008 | China | 69.4 | 56.2±10.61* | 1632 | 47.42±12.36* | 58.64 | Laminin | 31.7 | S |
| M-G Choi[22] | 2009 | Korea | 58.3 | 57.4(23-81.4) | 180 | 30.5(10.3-146.2) | 43.33 | HE | 43.13 | S±C |
| Ahmet Bilici [3] | 2010 | Turkey | 66.4 | 59(29-85) | 238 | 29.5(7.5-73) | 78.99 | HE | 75.6 | S |
| Xie honghu[23] | 2010 | China | 72.5 | 61.2(>60)^a^ | 178 | >60 | 66.29 | HE | 43.8 | S |
| Li Ping[24] | 2011 | China | 70.9 | 59.6(20-86) | 669 | 30(1-73) | 65.17 | HE | 9.27 | S |
| Jing Deng[25] | 2012 | China | 66.8 | 48.29(>60)^a^ | 293 | 57(2-84) | 65.41 | HE | 40.9 | S±C |
| Deniz Tural[26] | 2012 | Turkey | 70 | 58(22-90) | 866 | 58(22-90) | NA | HE | NA | S±C |
| Fatih Selcukbiricik[27] | 2012 | Turkey | 74 | 59(23-89) | 287 | NA | 67.6 | HE | 73 | S±C±R |
| Hsu-Huan Chou[28] | 2012 | Taiwan | 66.3 | 62.8±13.6* | 448 | 78.7(3.5-188.7) | 0 | HE | 33.03 | S±C |
| M-G Choi[29] | 2012 | Korea | 66.3 | 56.3±11.9* | 10728 | 40.7±29.9* | 36.46 | HE | 17.53 | S±C |
| Wilson L Costa Jr[30] | 2012 | Brazil | 57 | 63(21-88) | 142 | 45 | 73.24 | HE | 47.18 | S±C±R |

To be continued

Continued

| Author | Year | | Country | Male (%) | Age | N | Follow-up time(months) | LN(+) rate(%) | PNI detectionMethod | PNI positive rate(%) | Treatment |
| --- | --- | --- | --- | --- | --- | --- | --- | --- | --- | --- | --- |
| Woo-Sang RYU[31] | | 2012 | Korea | 60.5 | 63.5(>50)^b^ | 148 | 35.8(15-69) | 50.68 | HE | 8.04 | S |
| Dae Hoon Kim[32] | | 2013 | Korea | 66.1 | 55.3±12* | 7757 | 56.1±39.5* | 38.7 | HE | 27.7 | S±C±R |
| Jin Won Kim[33] | | 2013 | Korea | 65.4 | 60(28-87) | 318 | 71.8 | 67.3 | HE | 56.9 | S±C |
| K.A.Bickenbach[34] | | 2013 | U.S.A | 65.8 | 22-96 | 2384 | NA | 75 | HE | NA | S±C±R |
| Kyoung-Joo Kwon[35] | | 2013 | Korea | 64 | 60.9±12.4* | 769 | 105.05(0-152) | 49.15 | HE | 27.31 | S±C |

* Mean; a, percentage of aged over 60; b, percentage of aged over 50
