# Supplementary material for: Prognostic Value of Perineural Invasion in Gastric Cancer: A Systematic Review and Meta-Analysis
Source: PLoS One. 2014 Feb 21;9(2):e88907. doi: 10.1371/journal.pone.0088907 (PMC3931634; doi:10.1371/journal.pone.0088907)
Supplement: Table S2 — Statistical analysis data for OS of the studies. (DOCX) [file pone.0088907.s002.docx]

**Table S2.** Statistical analysis data for OS of the included studies.

| Study | Univariate analysis(unjusted)  HR 95% CI P value | | | Multivariate analysis(justed)  HR 95%CI P value | | |
| --- | --- | --- | --- | --- | --- | --- |
| Akira Tanaka 1993[14] | 2.672 | 1.962-3.638 | <0.0001 | - | - | - |
| Lp Setala 1996[15] | 1.43 | 1.22-1.675 | 0.000 | - | - | - |
| Chia-Siu Wang 2001[17] | 1.836 | 1.671-2.018 | <0.0001 | - | - | - |
| C Fondevila 2004[19] | 1.082 | 0.568-2.062 | <0.039 | 2.83 | 1.17-6.85 | <0.03 |
| M-G Choi 2009[22] | 1.771 | 1.304-2.405 | <0.001 | 1.76 | 1.06-2.9 | 0.027 |
| Xie honghu 2010[23] | 3.292 | 1.936-5.599 | <0.001 | 2.257 | 1.268-4.019 | 0.006 |
| Li Ping 2011[24] | 1.309 | 1.113-1.54 | <0.05 | - | - | - |
| Deng jing 2011[25] | 3.492 | 2.35-5.189 | 0.000 | 1.46 | 0.92-2.317 | 0.108 |
| Hsu-Huan Chou 2012[28] | 2.17 | 1.34-3.5 | 0.002 | 1.93 | 1.2-3.1 | 0.007 |
| M-G Choi 2012[29] | 3.126 | 2.891-3.379 | <0.001 | - | - | - |
| Wilson L Costa Jr 2012[30] | 1.703 | 1.13-2.567 | 0.015 | 2.03 | 1.17-3.53 | 0.011 |
| Woo-Sang RYU 2012[31] | 4.616 | 2.697-7.901 | <0.001 | 0.87 | 0.42-1.801 | 0.707 |
| Kyoung-Joo Kwon 2013[35] | 3.24 | 2.112-4.972 | <0.001 | 1.145 | 0.687-1.91 | 0.603 |
| Dae Hoon Kim 2013 [32] | 2.969 | 2.74-3.216 | <0.001 |  |  |  |
| Anna Maria Chiaravalli 2001[16] | - | - | - | 0.87 | 0.47-1.61 | 0.65 |
| L Setala 2001[18] | - | - | - | 1.57 | 1.1-2.26 | 0.014 |
| Nuvit Duraker 2002[4] | - | - | - | 1.1 | 0.89-1.37 | 0.352 |
| M Scartozzi 2006[20] |  |  |  | 0.7093 | 0.4599-0.9393 | 0.0402 |
| Luo tianhang 2008[21] |  |  |  | 3.23 | 2.6-8.11 | <0.01 |
| Ahmet Bilici 2010[3] | - | - | - | 2.75 | 1.12-3.13 | 0.02 |
| Fatih Selcukbiricik 2012[27] | - | - | - | 1.21 | 1.08-2.3 | 0.025 |
| Deniz Tural 2012[26] | - | - | - | 1.67 | 1.08-2.78 | 0.043 |
| K.A.Bickenbach 2013[34] | - | - | - | 1.3 | 1.1-1.6 | <0.001 |
